# Abnormalities in circulating plasmacytoid dendritic cells in patients with systemic lupus erythematosus

**DOI:** 10.1186/ar3075

**Published:** 2010-07-09

**Authors:** Ou Jin, Sushma Kavikondala, Mo-Yin Mok, Lingyun Sun, Jieruo Gu, Rong Fu, Albert Chan, Joseph Yeung, Yingjie Nie, Chak-Sing Lau

**Affiliations:** 1 Division of Rheumatology and Clinical Immunology, Department of Medicine, Queen Mary Hospital, The University of Hong Kong, 102 Pokfulam Road, Hong Kong, PR China; 2 Department of Rheumatology, Third Hospital of Yat-sen Sun University, 600 Tian He Road, Guang Zhou, 510630, PR China; 3 Department of Rheumatology, Drum Tower Hospital, The University of Nanjing, 321 Zhong Shan Road, Nanjing, 210008, PR China

## Abstract

**Introduction:**

Dendritic cells (DCs) are capable of inducing immunity or tolerance. Previous studies have suggested plasmacytoid DCs (pDCs) are pathogenic in systemic lupus erythematosus (SLE). However, the functional characteristics of directly isolated peripheral circulating blood pDCs in SLE have not been evaluated previously.

**Methods:**

Peripheral blood pDCs from 62 healthy subjects and 58 SLE patients were treated with apoptotic cells derived from polymorphonuclear cells (PMNs). Antigen loaded or unloaded pDCs were then co-cultured with autologous or allogenous T cells. Changes in T cell proliferation, cell surface CD25 expression, intracellular Foxp3 expression and cytokine production were evaluated. pDCs that had captured apoptotic PMNs (pDCs + apoPMNs were also studied for their cytokine production (interferon (IFN)-alpha, interleukin (IL)-6, IL-10, IL-18) and toll like receptor (TLR) expression.

**Results:**

Circulating pDCs from SLE patients had an increased ability to stimulate T cells when compared with control pDCs. Using allogenous T cells as responder cells, SLE pDCs induced T cell proliferation even in the absence of apoptotic PMNs. In addition, healthy pDCs + apoPMNs induced suppressive T regulatory cell features with increased Foxp3 expression in CD4 + CD25 + cells while SLE pDCs + apoPMNs did not. There were differences in the cytokine profile of pDCs that had captured apoptotic PMNs between healthy subjects and patients with SLE. Healthy pDCs + apoPMNs showed decreased production of IL-6 but no significant changes in IL-10 and IL-18. These pDCs + apoPMNs also showed increased mRNA transcription of TLR9. On the other hand, while SLE pDCs + apoPMNs also had decreased IL-6, there was decreased IL-18 mRNA expression and persistent IL-10 protein synthesis. In addition, SLE pDCs lacked TLR9 recruitment.

**Conclusions:**

We have demonstrated that peripheral circulating pDCs in patients with SLE were functionally abnormal. They lacked TLR9 expression, were less capable of inducing regulatory T cell differentiation and had persistent IL-10 mRNA expression following the capture of apoptotic PMNs. We suggest circulating pDCs may be pathogenically relevant in SLE.

## Introduction

Dendritic cells (DCs) are professional antigen presenting cells (APCs) with important immunoregulatory functions. They are the only cells that can stimulate naïve T cells [1]. Commonly, DCs circulate in peripheral tissues, capture pathogens or dying cells, and present antigens to T cells. T cells then proliferate and differentiate into Th1 (cell immunity), Th2 (humoral immunity) or T regulatory (Treg) (suppressive) cells resulting in the induction of immunity or tolerance [2]. Recent studies have shown that cytokines produced by DCs contribute to the induction of T cell differentiation [1]. For example, interleukin (IL)-6 primes CD4^+^T cells to differentiate into Th2 cells [3] and suppresses the activity of Treg cells [4]. IL-10 inhibits Th1 but induces Th2 responses [5]. IL-18, previously known as IFN-γ-inducing factor, interacts with IL-12 and induces naive T cell proliferation and differentiation into interferon (IFN)-γ producing Th1 cells [6].

Considering the role of DCs in the induction of immunity or tolerance in health, changes in DC function in autoimmune diseases such as systemic lupus erythematosus (SLE) which is characterized by loss of tolerance to self antigens have been studied intensively. Actually, DC functional abnormalities in SLE have been reported previously, the main progress of which is on the pathogenic role of plasmacytoid DCs (pDCs) in this condition [7]. Though the precise role of pDCs in the immune system is still unclear, these cells have been shown to polarize T cells through its high production of IFN-α [2] , which is generally considered to be the central cytokine that contributes to SLE development [8]. However, it is puzzling that some previous studies have reported diminished IFN-α production and T cell-stimulatory capacity by cytokine-induced pDCs in SLE [9]. Further, high serum levels of IFN-α are only found in some but not all SLE patients [10]. The role of pDCs in SLE requires further clarification.

Apoptotic cells are the primary source of autoantigens in SLE [11,12]. Nucleic-acid containing macromolecules which comprise the majority of autoantigens in SLE have been detected on the surface of apoptotic bodies [13]. The hypothesis is that in the process of apoptosis, nuclear antigens are cleaved into DNA fragments, revealing previously cryptic epitopes or neoepitopes to activate the immune system [12]. Supportive evidence comes from studies which showed DNA fragments isolated from sera of SLE patients stimulated mononuclear cell proliferation, and that these DNA fragments were of the same size as internucleosomal digested DNA from apoptotic cells [14].

Recently, an animal study has shown apoptotic cell-pulsed bone marrow-derived DCs (AC-BMDCs) could induce the proliferation of self reactive T-cells resulting in tolerance break down and initiation of autoimmune responses in normal mice [15]. However, until now no direct studies have been carried out in human SLE evaluating the function of peripheral blood pDCs and their interactions with immune cells following loading with apoptotic cells. In this study, we carried out experiments to examine the characteristics and functions of freshly isolated circulating pDCs from healthy and SLE subjects in the absence or presence of apoptotic cells. We hypothesize that peripheral circulating pDCs are functionally abnormal in SLE.

## Materials and methods

### Subjects

Patients who fulfilled the American College of Rheumatology criteria for SLE [16] were studied. There were 50 females and 8 males, with their age ranging from 21 to 62 (41.76 ± 9.15) years and disease duration from 1 to 28 (10.58 ± 7.16) years. Disease activity was assessed using the SLE disease activity index (SLEDAI) [17]. Active disease was defined as SLEDAI ≥ 5 (*n *= 26). Thirty-two patients had inactive disease (SLEDAI < 5). Sixty-two sex- and age-matched healthy volunteers were recruited from the Red Cross Blood Transfusion Section. The study was approved by the Hong Kong West Cluster Institutional Review Board for medical ethics. All subjects provided a written informed consent.

### An overview of the experimental design

(1) Circulating blood pDCs were isolated and cultured with or without apoptotic cells; (2) Antigen loaded or unloaded pDCs were then co-cultured with autologous or allogenous T cells. The same control T cells were used as responder cells in all allogenic proliferation assays. Changes in T cell proliferation, cell surface marker CD25 expression, CD4^+^CD25^+ ^Foxp3^+ ^expression and cytokine production were evaluated; and (3) functional changes of pDCs after interaction with apoptotic cells were evaluated by detection of cytokine production and toll-like receptor (TLR) expression.

### Blood collection and cell isolation

A total of 100 mls of sodium citrate anti-coagulated blood were collected between 9:00 and 11:00 AM. Peripheral blood mononuclear cells (PBMCs) were freshly isolated by Ficoll density gradient centrifugation. pDCs and T cells from PBMCs were magnetically sorted with the human BDCA-4 DC and pan T isolation kits respectively according to the manufacturer's description (Miltenyi Biotec, Berqisch Gladbach, Germany). Briefly, pDCs were positively selected using anti-BDCA-4 conjugated beads [18]. T cells were negatively isolated using magnetic beads conjugated with various surface antigen antibodies (anti-CD14, anti-CD16, anti-CD19, anti-CD36, anti-CD56, anti-CD123) to remove non-T cells. The purity of pDCs and T cells was approximately 90% and approximately 99%, respectively.

After isolating PBMCs from the whole blood, dextran sedimentation was applied to separate polymorphonuclear cells (PMNs, neutrophils) from red blood cells (RBCs). The remaining RBCs were lyzed with ammonium chloride (BD, CA) and removed by washing with saline. Purified PMNs were then resuspended with complete RPMI 1640 medium at a concentration of 5 × 10^6 ^cells/ml. PMNs were given 120 mJ/ml ultraviolet (UV) irradiation using the CL-100 Ultraviolet Crosslinker (Upland, CA, USA) to induce apoptosis. Sixteen hours after UV irradiation, the rate of apoptosis on PMNs reached around 60% to 90% and was confirmed by microscopic examination of cytocentrifuge stained with May-Giemsa, and flow cytometric detection of annexin V and propidium iodide (PI) staining (double positive cells).

### Interaction of dendritic cells with apoptotic cells

pDCs were incubated with apoptotic PMNs for four hours. pDCs that had captured apoptotic PMNs (pDCs + apoPMNs) were confirmed by flow cytometry detection of surface PE-Cy5 CD123 (for pDC) and 5-(and 6)-carboxytetramethylrhodamine succinimidyl ester (for PMNs) double positive stained cells, the percentage of which was around 15%. pDCs or pDCs + apoPMNs were then treated with mitomycin C, which has the ability to inhibit proliferation without affecting the viability of the feeder cells. The cell cultures were subsequently washed with phosphate buffer solution (0.5% BSA) to remove mytomycin C and apoptotic PMNs, for T cell interaction experiments or for evaluation of cytokine and TLR expression.

### Mixed leukocyte reaction (MLR)

T cell proliferation and differentiation induced by pDCs were evaluated by MLR. In brief, pDCs (1.0 × 10^4^) or pDCs that had interacted with apoptotic PMNs for four hours (pDC:PMN ratios = 1:1, 1:5, and 1:10) were used to stimulate autologous or allogenous responder T cells (1.0 × 10^5^). After five days of culture in a 96-well round-bottom culture plates with 200 μl of complete RPMI 1640 culture medium, the culture supernatants were collected for the detection of cytokines produced by T cells, and fresh medium was added back. Following that, 0.5 μCi tritiated thymidine (Radiochemical Centre, Amersham, Little Chalfont, Buckinghamshire, UK) was added per well for 16 hours. T cell proliferation was measured by detection of the incorporation of (3H)-thymidine. Cells were harvested with a Packard FilterMate™ Universal Harvester (Downers Grove, IL, USA) and read with a TopCount™ NXT Microplate Scintillation Counter (Perkin Elmer, Boston, MA, USA). After the initial experiments, we found the 1:10 pDC:PMN ratio resulted in maximum T cell stimulation. This ratio was used in later experiments.

### Flow cytometry

The purity of pDCs and T cells was confirmed by surface immunofluorescence staining and flow cytometry analysis. pDCs were defined as BDCA-2^+^CD123^+^lin^- ^cells [19] and its purification was above 90% [18]. Comparable levels of surface markers expression including MHC-II CD86 CD83 and CCR7 were found in pDCs from both SLE and healthy [18]. T cells were defined as CD3^+ ^cells and its purification is about 99% (data not shown). Changes in surface CD25 expression of T cells stimulated by pDCs were measured by percentage of CD4^+^CD25^+ ^cells per CD4^+ ^T cells and compared with that of unstimulated T cells. To further investigate whether the CD4^+^CD25^+ ^T cells were Treg cells or not, intracellular immunofluorescence staining of Foxp3 was carried out according to the manufacturer's description (eBioscience, San Diego, CA, USA). Appropriate isotype-matched control Ig was used as negative controls for each analysis. Flow cytometric analysis was performed within 24 hours, on a FACSCalibur flow cytometry using Cellquest software.

### ELISA

Cytokines produced by T cells after interaction with pDCs or pDCs + apoPMNs were measured using OptEIATM ELISA (BD Biosciences Pharmingen, San Diego, CA, USA). Supernatants in the MLR system were collected after five days culture and frozen at -70°C for subsequent cytokine detection. These cytokines included IFN-γ, tumor necrosis factor (TNF)-α and IL-2 (Th1 cytokines); IL-4 and IL-6 (Th2 cytokines); and IL-10 and transfer growth factor (TGF)β (Treg cytokines) [2].

Changes in cytokine production by pDCs or pDCs + apoPMNs were evaluated also using ELISA. Supernatants from the pDC culture system were collected after 24 hours' culture and frozen at -70°C for subsequent detection of IFN-α, IL-6, IL-10, IL-12, TNF-α levels. Human IFN-α ELISA kit was obtained from PBL Biomedical Laboratory (Piscataway, NJ, USA). ELISA results were read using MQX200 FCC Compliance (BID-TEK Instruments, Inc., Sacramento, CA, USA).

### Real time polymerase chair reaction (PCR)

Semi-quantitative real time PCR was applied to detect changes in cytokine or TLR mRNA gene expression in pDCs after interaction with apoptotic cells for four hours. pDCs without interaction with apoptotic PMNs and PMNs alone were used as controls. Total RNA from the culture system was isolated through PurelinkTM Micro-to-Midi Total RNA Purification System (Invitrogen, Carlsbad, CA, USA) according to the manufacturer's description. First-strand complementary DNA was subsequently synthesized using the SuperScriptTM First-Stand Synthesis System (Invitrogen). For real-time PCR detection of target and housekeeping gene expression, the fluorescent TaqMan 5'-nuclease assay was performed using 2× TaqMan Universal PCR Master Mix and TaqMan Gene Expression assays (two unlabeled primers and one 6-FAM or VIC dye -labeled TaqMan MGB probe) for IL-6 (Hs00174131-ml), IL-10 (Hs00174086-ml), IL-18 (Hs00155517-ml), IFN-α1 (Hs00256882-sl), TLR-4 (Hs00152939-ml), TLR-7 (Hs00152971-ml), TLR-9 (Hs00370913-sl) and 18S (Hs999999901-sl) (Applied Biosystems, Foster City, CA, USA). The reaction was performed in triplicate on an ABI 7000 Sequence Detector (Applied Biosystems) with a standard run. The expression levels of the target genes were adjusted to that of the housekeeping genes 18S. The fold change in the pDC target gene expression was adjusted to that of 0 hour, represented as the 2^-ΔΔCt^, where ΔΔCt = (Ct.target - Ct.18S)_Time.x _- (Ct.target - Ct.18S)_Time.0h_.

### Statistical analysis

As this was the first study on human peripheral circulating pDCs, the data were first tested for normality before statistical analysis to detect group differences was carried out. Normally distributed data are expressed as mean ± SD while non-normally distributed data as median (interquartile range). For comparison across different groups, a two-factor analysis of variance (ANOVA) was used for normally distributed data. In case the ANOVA indicated differences across groups (*P *< 0.10), a pair-wise Student's *t*-test was used to evaluate the explicit *P*-values. Similarly, the Kruskal-Wallis test was used to compare across different groups of non-normally distributed data. If differences were indicated across groups, a Wilcoxon's rank sum test for two samples was used to evaluate the explicit *P*-values.

## Results

### T cell proliferation stimulated by pDCs or pDCs + apoPMNs

#### Autologous MLR T cell proliferation

**Figure 1:**
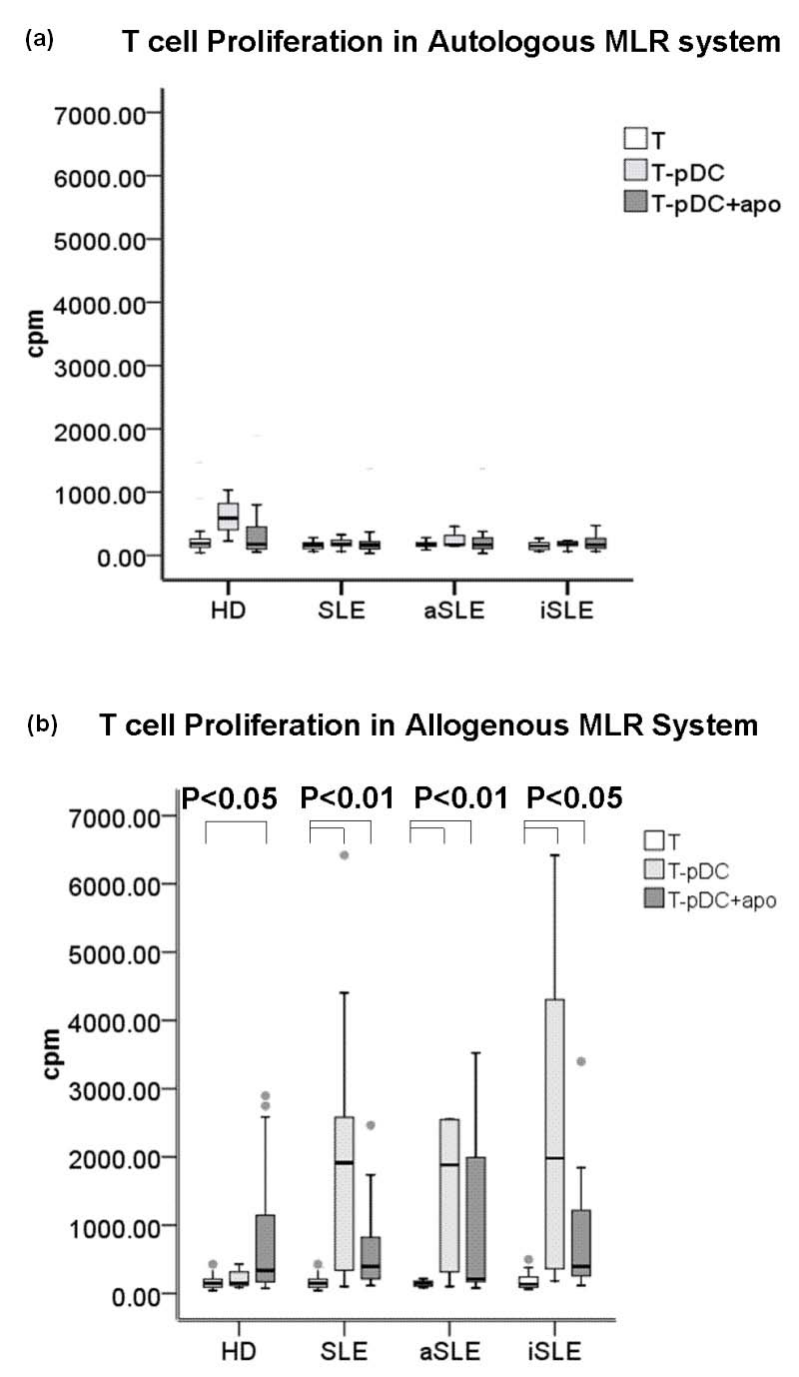
**T cell proliferation following stimulation with pDCs alone or pDCs + apoPMNs in the MLR systems**. **(a) **In the autologous MLR system: pDCs from healthy donors (*n *= 36) did not induce T cell proliferation, regardless of loading with apoptotic PMNs or not. The same phenomenon was also found in SLE patients (*n *= 30; 12 with active disease and 18 with inactive disease), irrespective of disease activity status. **(b) **In the allogenous MLR system: In healthy donors (*n *= 36), only pDCs + apoPMNs induced allogenous T cell proliferation. With SLE pDCs, both pDCs alone and pDCs + apoPMNs induced allogenous T cell proliferation, suggesting a higher ability of SLE pDCs to stimulate allogenous T cells. Values are expressed in Median (interquartile range, range). auto-T: autologous T cells, allo-T: allogenous T cells, pDCs + apoPMNs: pDCs that had been loaded with apoptotic polymorphonuclear cells. HD, healthy donors; aSLE, active SLE; iSLE, inactive SLE

pDCs from healthy donors (*n *= 36) did not induce autologous T cell proliferation whether they were fed with apoptotic cells or not (Figure 1a). Similarly, SLE (*n *= 30) pDCs did not stimulate autologous T cell proliferation, irrespective of the overall SLE disease activity (Figure 1a).

#### Allogenous MLR T cell proliferation

In the allogenous MLR system, only pDCs + apoPMNs from healthy subjects (*n *= 36) induced allogenous T cell proliferation while both pDCs and pDCs + apoPMNs from SLE patients (*n *= 30) were able to induce T cell proliferation (Figure 1b). As pDCs have a high rate of apoptosis themselves [20] (approximately 40%, unpublished data), pDCs alone cultures may be regarded as a system of low level self-antigen presentation. These results suggest SLE pDCs have a higher ability to stimulate T cells than controls. This was observed irrespective of the patients' disease activity.

### CD25 and Foxp3 expression on CD4^+ ^T cells

**Figure 2:**
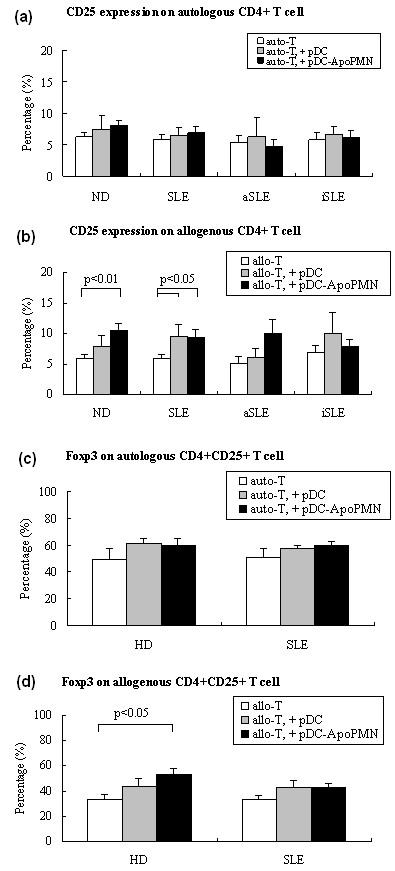
**CD4 + T cell CD25 and Foxp3 expression following incubation with pDCs or pDCs + apoPMNs in MLR systems**. **(a) **Autologous MLR system T cell CD25 expression: No changes were detected on CD4 + T cells from either healthy subjects (*n *= 36) or patients with SLE (*n *= 30; 12 with active disease and 18 with inactive disease) following incubation with pDCs alone or pDCs + apoPMNs. Values are expressed in Median (interquartile range, range). **(b) **Allogenous MLR system T cell CD25 expression: CD25 expression was increased following incubation with healthy pDCs + apoPMNs (*n *= 36) but not pDCs alone, whereas a significant increase was seen following incubation with both SLE pDCs alone and pDCs + apoPMNs (*n *= 30). Values are expressed in Median (interquartile range, range). **(c) **Autologous MLR system CD4 + CD25 + T cell intracellular staining of Foxp3: No changes were detected following incubation with either pDCs alone or pDCs + poPMNs from healthy subjects (*n *= 6) and patients with SLE (*n *= 6). Values are expressed in Mean ± SEM. **(d) **Allogenous MLR system CD4 + CD25 + T cell intracellular staining of Foxp3: pDCs + apoPMNs from healthy donors increased the expression of Foxp3 on allogenous CD4 + CD25 + T cells (*n *= 8, *P *< 0.05), but no significant changes were seen with SLE pDCs alone or pDCs + apoPMNs (*n *= 8). Values are expressed in Mean ± SEM. auto-T: autologous T cells, allo-T: allogenous T cells, pDCs + apoPMNs: pDCs that had been loaded with apoptotic polymorphonuclear cells. HD, healthy donors; aSLE, active SLE; iSLE, inactive SLE.

In the autologous MLR system, neither healthy nor SLE pDCs or pDCs + apoPMNs increased CD25 expression on CD4^+ ^T cells (Figure 2a). In the allogenous MLR system, both healthy and SLE pDCs + apoPMNs induced CD25 expression on CD4^+ ^T cells (Figure 2b). Consistent with the proliferation assay results, SLE pDCs alone increased CD25 expression on allogenous CD4^+ ^T cells (Figure 2b), indicating that SLE pDCs had a higher ability to stimulate CD25 expression on allogenous CD4^+ ^T cells.

To evaluate whether the CD25^+^CD4^+ ^T cells may represent either active effector T cells or immunosuppressive Treg cells [21], intracellular staining and flow cytometry detection of Foxp3, Treg cell signatory, [22] were carried out. The percentage of CD4^+^CD25^+ ^T cells that were Foxp3^+ ^was evaluated. Neither healthy nor SLE pDCs changed the expression of Foxp3 in autologous CD4^+^CD25^+ ^T cells (Figure 2c). With allogenous CD4^+^CD25^+ ^T cells, however, healthy pDCs + apoPMNs increased the expression of Foxp3 (Figure 2d, *P *< 0.05) while no significant changes were found with either SLE pDCs alone or pDCs + apoPMNs. These results suggest that healthy but not SLE pDCs induced the development of suppressive Treg cells.

### T cell cytokine production

**Figure 3:**
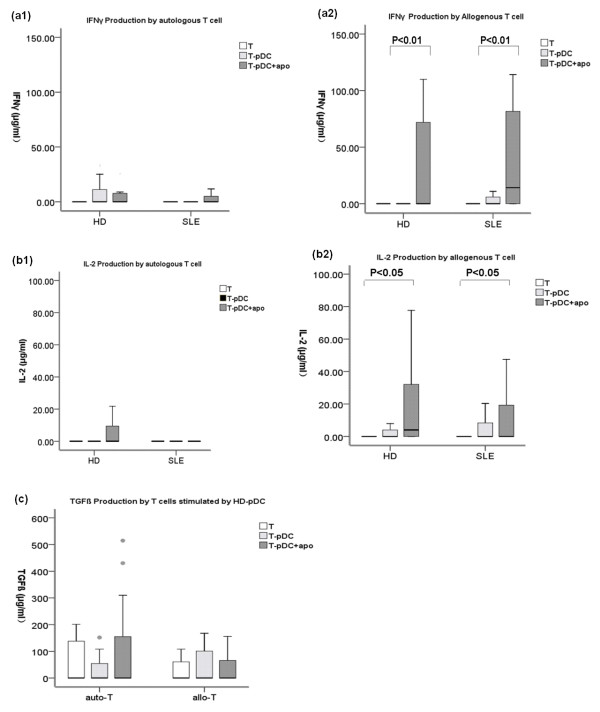
**T cell production of IFNγ, IL-2 and TGFβ following incubation with pDCs alone and pDCs + apoPMNs**. **(a1) **and **(b1) **Autologous T cells that had been incubated with either healthy donor (*n *= 30) or SLE (*n *= 30) pDCs alone or pDCs + apoPMNs did not produce Th1 related cytokines IFNγ and IL-2. **(a2) **and **(b2) **Allogeneous T cells that had been incubated with either HD (*n *= 30) or SLE (*n *= 30) pDCs + apoPMNs produced IFNγ and IL-2. **(c) **Autologous and allogeneous T cells that had been incubated with either HD (*n *= 30) pDCs alone or pDCs + apoPMNs produced low levels of Treg related TGFβ though the difference did not reach statistical significance. Values are expressed in Median (interquartile range, range). auto-T, autologous T cells; allo-T, allogenous T cells; pDCs + apoPMNs, pDCs that had been loaded with apoptotic polymorphonuclear cells; HD, healthy donors.

Neither healthy nor SLE pDCs or pDCs + apoPMNs induced autologous T cells to produce Th1 (Figure 3a1, Figure 3b1) or Th2 related cytokines (data not shown). In the allogenous MLR system, both healthy and SLE pDCs + apoPMNs induced Th1 cell related cytokines including IFNγand IL-2 (Figure 3a2, 3b2).

Both autologous and allogenous T cells that were co-cultured with healthy pDCs produced a small amount of Treg cell related TGFβ (Figure 3c) but the changes were not statistically significant.

### Changes in the functional characteristics of pDCs + apoPMNs

#### pDC cytokine production

**Figure 4:**
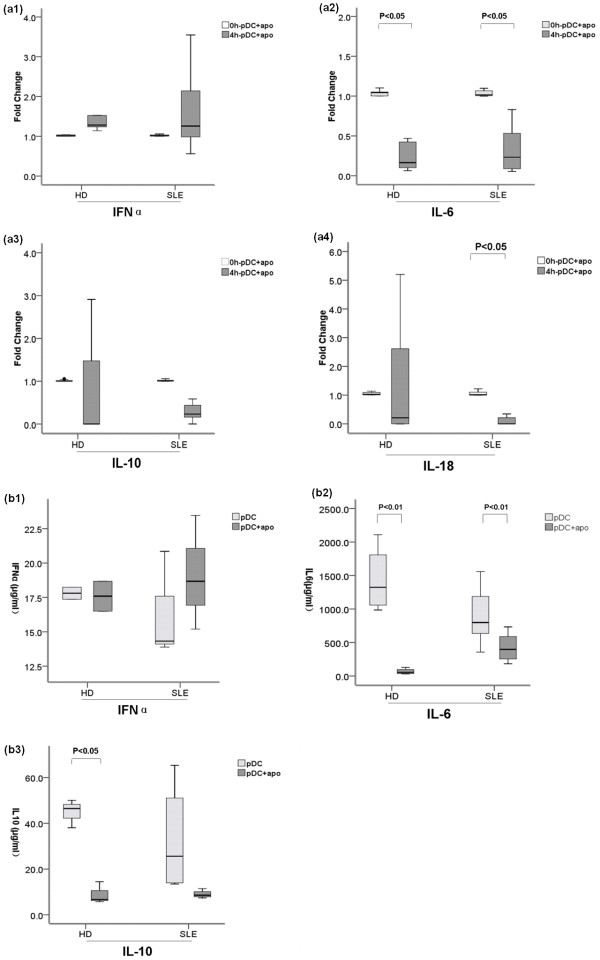
**Changes in mRNA expression and protein levels of cytokines in HD and SLE pDCs + apoPMNs**. **(a) **Changes in IFNα, IL-6, IL-10 and IL-18 mRNA expression in HD (*n *= 7) and SLE (*n *= 7) pDCs + apoPMNs. IFNα mRNA expression (A1) appeared increased in both HD and SLE pDCs + apoPMNs but the changes did not reach statistical significance. IL-6 (A2) mRNA expression was significantly decreased in both HD and SLE pDCs + apoPMNs. IL-10 mRNA expression (A3) did not change in both HD pDCs + apoPMNs. IL-18 mRNA expression (A4) did not change in HD pDCs + apoPMNs but was significantly decreased in SLE pDCs + apoPMNs. **(b) **protein levels of IFNα, IL-6 and IL-10 in the supernatants of cultured HD (*n *= 8) and SLE (*n *= 8) pDCs + apoPMNs (B1, B2 and B3). Results were compared with cultured pDCs alone. B1) No significant differences in the levels of IFNα in the culture supernatants of both HD and SLE pDCs + apoPMNs were detected. B2) Levels of IL-6 were significantly decreased in the culture supernatants of both HD and SLE pDCs + apoPMNs. B3) Levels of IL-10 in the culture supernatants of HD pDCs + apoPMNs were significantly decreased while no significant changes were found in that of SLE pDCs + apoPMNs. Values are expressed in Median (interquartile range, range). pDCs + apoPMNs, pDCs that had been loaded with apoptotic polymorphonuclear cells; HD, healthy donors; IFNα, Interferon α; IL, Interleukin.

Following incubation with apoptotic cells for four hours, both healthy and SLE pDCs showed a slight increase in the expression of IFNα mRNA but it did not reach statistical significance (Figure 4a1). Both healthy and SLE pDCs + apoPMNs had decreased mRNA expression of IL-6 (Figure 4a2). There were no significant changes in the IL-10 mRNA expression of both healthy and SLE pDCs + apoPMNs (Figure 4a3). IL-18 mRNA expression in normal pDCs + apoPMNs did not change significantly but SLE pDCs - apoPMNs had decreased IL-18 mRNA expression (Figure 4a4).

Results of the ELISA of cytokines in supernatants of pDCs + apoPMNs cultures were in accordance with mRNA expression findings above. There were no changes in the level of IFNα from healthy and SLE pDCs + apoPMNs (Figure 4b1). IL-6 production by healthy and SLE pDCs + apoPMNs was both decreased (Figure 4b2). Interestingly, however, IL-10 production by healthy pDCs + apoPMNs was reduced but remained unchanged in SLE pDCs + apoPMNs (Figure 4b3).

#### pDC TLR expression

**Figure 5:**
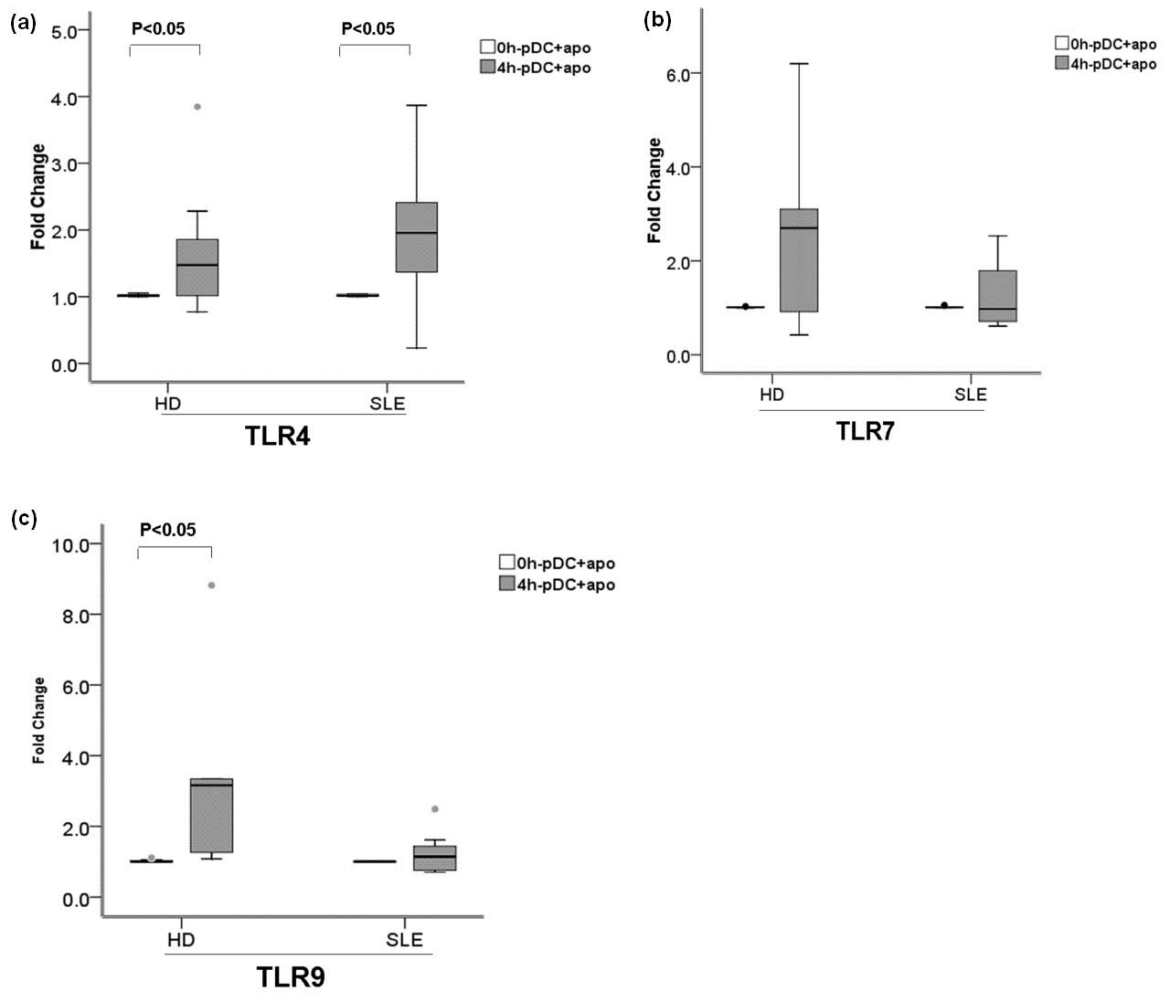
**TLR4, TLR7 and TLR9 expression on HD and SLE pDCs + apoPMNs**. HD (*n *= 5) pDCs + apoPMNs showed a significant increase in the expression of TLR9 mRNA **(c) **while SLE (*n *= 5) pDCs + apoPMNs did not. No significant changes in TLR4 **(a) **and TLR7 **(b) **expression were found in either HD or SLE pDCs + apoPMNs. Values are expressed in Median (interquartile range, range). pDCs + apoPMNs, pDCs that had been loaded with apoptotic polymorphonuclear cells; HD, healthy donors.

TLR9 mRNA expression in healthy pDCs + apoPMNs was found to be increased (Figure 5c) (*P *< 0.05). This was not observed with SLE pDCs + apoPMNs. No significant differences in the changes in TLR7 and TLR4 mRNA expression in between healthy and SLE pDCs + apoPMNs were detected (Figure 5a, b).

## Discussion

Previous reports have shown pDCs induce immune tolerance after phagocyting apoptotic cells [23]. However, other studies have also suggested that an increased number of apoptotic cells in the circulating blood are associated with autoimmunity [10,24]. Thus, we designed the current series of experiments to investigate the capacity of apoptotic cell loaded pDCs in stimulating T cells. To our knowledge, this is the first study which evaluated the effects of directly isolated peripheral pDCs on T cells in healthy subjects and patients with SLE. Our results show that healthy pDCs + apoPMNs did not induce autologous T cell proliferation (Figure 1a). This lack of ability of pDCs to stimulate self T cells even when they encountered apoptotic cells suggests that pDCs may play a tolerance role in immunity in health. Indeed, previous animal studies have also suggested a tolerance role of pDCs [25]. In humans, pDCs have been found to elicit antigen-specific anergy in CD4^+ ^T cell lines [26]. Furthermore, monocty-derived DCs were reported to be poor stimulators of T cells [25]. Our previous study on human circulating pDCs also suggested a low stimulatory capacity of pDCs as evidenced by their low level of co-stimulatory surface molecule expression [18].

We also investigated the ability of pDCs to stimulate allogenous T cells as an assessment of the antigen-presenting capability of DCs [27]. We found that healthy pDCs + apoPMNs induced only a very low level of allogenous T cell proliferation (Figure 1b) while SLE pDCs were more capable of inducing allogenous T cell proliferation (Figure 1b). Some previous studies have shown the opposite results. For example, DC-enriched APCs from SLE patients have been shown to have diminished T cell stimulatory capacity [28], or SLE peripheral DCs had reduced capacity to induce concanavalin A (Con A)-stimulated T cell proliferation [29]. However, these studies did not use purified DCs and their functions may be altered by other cells that were present in the assay.

In accordance with the proliferation results above, allogenous CD4^+ ^T cells that were co-cultured with either healthy or SLE pDCs + apoPMNs were found to have an increased expression of CD25 (Figure 2b). CD4^+^CD25^+ ^T cells may consist of either specific Th or Treg cell subsets [21]. As Foxp3 is now regarded as the specific signature of Treg cells [22], we evaluated the expression of Foxp3^+ ^in allogenous CD4^+^CD25^+ ^T cells induced by pDCs, only healthy pDCs + apoPMNs were found to have increased Foxp3 expression (Figure 2d), indicating the development of Treg cells. This is consistent with a recent mouse model which showed that murine pDCs that had acquired alloantigens from allografts during transplant mediated antigen-specific Treg cell development and allograft tolerance [30]. Studies on human pDCs have also revealed that CpG-oligodeoxynucleotides (ODN) stimulated circulating pDCs were able to induce Treg cell expansion [31]. Treg cells maintain peripheral tolerance by suppressing the activation and population expansion of self-reactive T cells [32].

Contrary to healthy pDCs - apoPMNs, SLE pDCs + apoPMNs did not induce Foxp3^+ ^expression in CD4^+^CD25^+^T cells (Figure 2d). The important role of Foxp3^+^CD4^+^CD25^+ ^Treg cells in immune homeostasis is that it controls autoimmunity throughout life. In animal models, Treg cell removal by neonatal thymectomy causes the spontaneous development of various organ-specific autoimmune diseases [33,34]. Therefore, the lack of capacity of SLE pDCs to induce Treg development may contribute to the break down of immune tolerance and the development of autoimmunity. Indeed, SLE patients have been found to have significantly lower numbers of CD4^+^CD25^+ ^T cells than normal persons [35]. In addition, a decreased suppressive function of CD4^+^CD25^high ^Treg cells with reduced level of Foxp3 mRNA and protein expression has also been found in active SLE [36]. Recently, a study has shown that APCs from SLE patients were responsible for decreased Treg cell activity though the authors had used non-T cells as APCs [37].

An important feature that differentiates the various subsets of T cells is the characteristic profile of cytokines produced by these cells [38,39]. Our results showed that both healthy and SLE pDCs + apoPMNs stimulated T cells produced Th1 related IFNγ and IL-2 (Figure 3a2, 3b2). Only healthy pDCs + apoPMNs stimulated T cells produced Treg2 cell-related TGFβ (Figure 3c) though the difference did not reach statistical significance. However, this latter finding provides further support of the notion that healthy pDCs + apoPMNs induce tolerogenic Treg cell expansion while SLE pDCs + apoPMNs do not.

Functional changes of pDCs + apoPMNs including cytokine production and TLR expression [40] were studied to delineate the probable mechanisms of these pDCs' effects on T cells. We found that circulating healthy pDCs + apoPMNs had decreased mRNA expression and protein levels of IL-6 (Figure 4a2) and protein levels of IL-10 (Figure 4B3). SLE pDCs + apoPMNs were also found to have decreased mRNA expression and protein levels of IL-6 (Figure 4a2). However, we also noted a decrease in IL-18 mRNA expression (Figure 4a4) and no significant changes in IL-10 protein levels in the supernatant of the SLE pDCs + apoPMNs cultures. These findings are interesting as DC IL-18 is involved in the induction of Th1 response and IL-10 induces Th2 response during interactions with T-cells. Our findings are in accordance with the notion that SLE is characterized by a Th1/Th2 imbalance toward Th2 dominance.

TLRs play an important role in the presentation of antigens derived from apoptotic cells by DCs [40-43]. In our study, we found that healthy pDCs + apoPMNs increased their expression of TLR9 mRNA (Figure 5c) and TLR4 mRNA (Figure 5a) while TLR7 (Figure 5b) mRNA expression remained unchanged (Figure 5a). A previous study has reported that human pDCs activated by the TLR9 ligand CpG-ODN could induce CD4^+^CD25^+ ^Treg cells [31]. With SLE pDCs + apoPMNs, however, no increase in TLR9 mRNA expression was found (Figure 5c). This is consistent with data presented in a previous study which found that the expression of TLR9 on SLE pDCs stimulated by CpG ODN decreased rapidly [44]. We hypothesize that this difference in TLR9 recruitment between SLE and healthy pDCs may partly contribute to the lack of induction of Treg cells development in SLE, and this may further contribute to the tolerance break-down and autoimmune disease development.

A limitation of this study is that most of the patients were receiving some form of treatment including immunosuppressive agents. It is therefore not possible to confirm whether the pDC changes were a result of the underlying disease or that of the various lupus medications. It should, however, be noted that differential changes were seen in patients with different levels of lupus disease activity. In general, the more active the disease was, the more adverse pDC changes were noted. It is therefore tempting to suggest that our findings reflect the true role of pDCs in lupus disease pathogenesis. Future studies should aim to recruit treatment of naïve or newly diagnosed patients with SLE. However, this will have to involve the collaboration of multiple lupus research units. It has taken the authors over two years to recruit 58 suitable patients from a cohort of over 500 patients for the purpose of this study.

## Conclusions

Our study has provided further insights into the role of pDCs in SLE. In patients with SLE, the capacity of circulating pDCs to stimulate T cells was increased while their ability to induce Treg cell development was decreased. These may be the results of decreased IL-18 and increased IL-10 transcription which may prime Th2 response, and low expression of TLR9 following pDCs' interaction with apoptotic cells.

## Abbreviations

AC-BMDCs: apoptotic cell-pulsed bone marrow-derived DCs; APCs: antigen presenting cells; DCs: dendritic cells; IFN: interferon; IL, interleukin; ODN: oligodeoxynucleotides; PBMCs: peripheral blood mononuclear cells; PCR: real time polymerase chair reaction; pDCs: plasmacytoid DCs; pDCs: + apoPMNs, pDCs: that had captured apoptotic PMNs; PMNs: polymorphonuclear cells; RBCs: red blood cells; SLE: systemic lupus erythematosus; SLEDAI: SLE disease activity index; TGF: transfer growth factor; TLR: toll like receptor; TNF: tumor necrosis factor; Treg: T regulatory; UV: ultraviolet.

## Competing interests

The authors declare that they have no competing interests.

## Authors' contributions

OJ carried out the whole study, performed the statistical analysis and drafted the manuscript. SK, RF and YN participated in some of the cell isolation and interaction assays. MYM participated in patient recruitment. LS and JG helped to draft the manuscript and performed the statistical analysis. AC carried out the flow cytometry, ELISA and MLR assays. JY participated in real time PCR detection. CSL conceived, participated in the design of and sought funding for the study. He coordinated patient recruitment and helped draft the manuscript. All authors read and approved the final manuscript.
